# *In Situ* Proteolysis to Generate Crystals for Structure Determination: An Update

**DOI:** 10.1371/journal.pone.0005094

**Published:** 2009-04-07

**Authors:** Amy Wernimont, Aled Edwards

**Affiliations:** Midwest Center for Structural Genomics and Structural Genomics Consortium, University of Toronto, Toronto, Ontario, Canada; Institute of Molecular and Cell Biology, Singapore

## Abstract

For every 100 purified proteins that enter crystallization trials, an average of 30 form crystals, and among these only 13–15 crystallize in a form that enables structure determination. In 2007, Dong *et al* reported that the addition of trace amounts of protease to crystallization trials—*in situ* proteolysis—significantly increased the number of proteins in a given set that produce diffraction quality crystals. 69 proteins that had previously resisted structure determination were subjected to crystallization with *in situ* proteolysis and ten crystallized in a form that led to structure determination (14.5% success rate). Here we apply *in situ* proteolysis to over 270 new soluble proteins that had failed in the past to produce crystals suitable for structure determination. These proteins had produced no crystals, crystals that diffracted poorly, or produced twinned and/or unmanageable diffraction data. The new set includes yeast and prokaryotic proteins, enzymes essential to protozoan parasites, and human proteins such as GTPases, chromatin remodeling proteins, and tyrosine kinases. 34 proteins yielded deposited crystal structures of 2.8 Å resolution or better, for an overall 12.6% success rate, and at least ten more yielded well-diffracting crystals presently in refinement. The success rate among proteins that had previously crystallized was double that of those that had never before yielded crystals. The overall success rate is similar to that observed in the smaller study, and appears to be higher than any other method reported to rescue stalled protein crystallography projects.

## Introduction

The field of protein crystallography has seen great progress in crystallization, data collection, phasing techniques, crystallization screens, robotics, as well as in software for data reduction, phasing, model building and refinement [1]. However, the overall success rate (as measured by the number of deposited structures per number of selected targets) remains relatively low [2], [3]. On average, only about 15–20% of protein targets that can be purified will then crystallize in a form from which a structure can be determined (http://www.targetdb.pdb.org; http://thesgc.com/structures/target_progress.php).

The field of protein crystallization, which previously focused almost entirely on the optimization of crystallization strategies, is now increasingly addressing the improvement of the crystallization properties of the proteins themselves [4]. This trend began in the early 1990's, with the advent of molecular biology techniques and mass spectrometry. The use of these techniques allowed scientists to focus crystallization efforts on the most stable domains of target proteins, as identified by their pattern of resistance to limited proteolysis [5], [6]. Stable domains crystallize more readily and often result in better-diffracting crystals [7], [8], [5]. Success rates were further increased by expressing many variations of the protein domain, as differences of a few residues at the N- or C- termini often have dramatic effects on soluble protein expression and protein crystallization. Graslund *et al.* found that by screening ten derivatives of a given protein domain instead of one, the probability of generating a soluble protein increased two-fold and the probability of generating a structure increased four-fold [9].

Crystallization can also be promoted by changing the surface properties of the protein to reduce the conformational entropy of surface residues. The most straight-forward approach is to use reductive methylation of surface lysine residues [10]. In large, systematic studies, lysine methylation rescued 6% of a set of recalcitrant proteins [11], [10]. Surface entropy can also be reduced by site-directed mutagenesis of clusters of charged residues [12]. Protein crystallization can also be facilitated by the addition of specific ligands or inhibitors, which bind to the protein and lower its intrinsic heterogeneity [13]. Finally, the addition of trace amounts of protease to the crystallization trials – *in situ* proteolysis – rescued 10 out of 69 different proteins (∼14%) that had previously failed in crystallization and structure determination [14].

*In situ* proteolysis appears to be the most efficacious crystallization rescue strategy. However, while the study of Dong *et al.* was systematic and rigorous, all but one of the successful cases derived from a single experimenter, and the proteins were predominantly of bacterial origin. This prompted further inquiry into whether the method (1) would be applicable to human proteins, (2) would be as successful in other hands, and (3) would be useful for those proteins for which dozens of variants had already been tested. This paper describes an expanded study: applying *in situ* proteolysis to 270 new proteins since the last paper, from prokaryotes and eukaryotes, and by dozens of scientists.

## Methods

All protein constructs contained an N-terminal hexahistidine tag, with a recognition site for TEV protease (MGSSHHHHHHSSGRENLYFQG or MGSSHHHHHHSSGRENLYFQGH or MAPEHHHHHHDYDIPTTENLYFQGA). Proteins were purified as described earlier [14], [15], [9]. All structures reported here have been deposited into the Protein Data Bank (PDB, www.rcsb.org).

Proteases were ordered from Sigma-Aldrich and stock solutions made up as follows: α-chymotrypsin (C3142) was dissolved in 1 mM HCl and 2 mM CaCl_2_ at a concentration of 1 mg/mL. Trypsin (T8003) was dissolved into 1 mM HCl and 2 mM CaCl_2_ at a concentration of 1.5 mg/mL. Elastase (E0127) was dissolved into 200 mM Tris-HCl buffer (pH 8.8) at a concentration of 1 mg/mL. Papain (P5306) was dissolved into water at a concentration of 1.2 mg/mL. Subtilisin A (P5380) was dissolved into 10 mM Na acetate and 5 mM Ca acetate at a concentration of 1 mg/mL. Endoproteinase Glu-C V8 (V8 protease) was dissolved into water at a concentration of 2 mg/mL. Stock solutions of protease were serially diluted into a buffer comprising 10 mM HEPES (pH 7.5) and 500 mM NaCl as needed.

### *In situ* proteolysis

*In situ* proteolysis was performed essentially as described [14], with the working set of proteases expanded, on a case-by-case basis, to include trypsin (at a range of 1∶10000 to 1∶10 v/v), V8 protease (at a range of 1∶100 to 1∶40 v/v), papain (at a range of 1∶1200 to 1∶10 v/v), thermolysin (at 1∶20 v/v), and subtilisin (at 1∶20 v/v).

### Pre-screening to identify a promising protease

Investigative limited proteolysis with a panel of proteases was used occasionally to identify a protease that generated promising degradation patterns, as detected by denaturing gel electrophoresis or mass spectrometry. 5–10 µL of protein, dissolved at 10–20 mg/mL, was incubated with a range of proteases for thirty minutes at room temperature. The proteases were used at dilutions of 1∶10, 1∶100, and 1∶1000. The reactions were stopped by the addition of SDS-Coomassie sample loading buffer for analysis by gel electrophoresis, or by formic acid for analysis by mass spectrometry. The protease(s) and concentration that yielded the largest, most stable domain were chosen for subsequent crystallization experiments.

## Results and Discussion

More than 270 proteins that failed to produce crystals or that produced crystal forms unsuitable for structure determination were subjected to *in situ* proteolysis crystallization trials over an eight-month period in 2008. This set comprised about 200 yeast or bacterial proteins, 70 human proteins, and 5 parasitic proteins. Of these, 34 proteins generated crystals of sufficient quality for structure determination (Table 1), for a rescue rate of ∼13%. 10 additional crystals in the set are being optimized, so the number of deposited structures may increase. We did not identify the protein cleavage sites using mass spectrometry, as was done in Dong et al, because of the effort involved. To indicate the approximate extent of cleavage, we have included in the Supplementary Information the sequence of each protein construct that entered crystal trials and the regions of the protein for which electron density was absent (Text S1).

**Table 1:** List of deposited protein targets crystallized by *in situ* proteolysis.

Target Name	Number of Constructs Designed	PDB Code	Crystal before protease	Resolution After Protease	Protease
CGD6_3220	20	**2RHD**	No crystal	2.1	T
KIF22	22	**3BFN**	No crystal	2.3	V8
EIF4A2	10	**3BOR**	No crystal	1.9	C
EIF3J	12	**3BPJ**	No crystal	1.9	C
RASL12	4	**3C5C**	Poor crystal	1.9	V8
EPHA2	22	**3C8X**	Poor crystal	2.0	T
REM2	8	**3CBQ**	low resolution	1.8	T
BB3683_1_259	1	**3CNV**	No crystal	2.0	T
NRAS	3	**3CON**	low resolution	1.7	C
NR1D2-07	17	**3CQV**	No crystal	1.9	T
TM1086	1	**3DCL**	large unit cell	2.3	C
SETDB1	36	**3DLM**	No crystal	1.8	T
ATC2088_1_254	1	**3DNH**	Poor crystal	1.9	T
APC85800.6	9	**3DOA**	No crystal	2.8	C
AF0924_65_195	1	**3DT5**	low resolution	1.9	T
TA0507_1_224	1	**3DTZ**	Poor crystal	1.8	C
TBC1D22B	6	**3DZX**	low resolution	2.3	C
DDB1	1	**3E0C**	No crystal	2.4	C
ARHGAP11A	24	**3EAP**	No crystal	2.3	V8
TA0454	1	**3EEF**	No crystal	2.4	T
APC7436	1	**3EET**	Twinned	2.0	Therm
XCC1504	2	**3EFG**	No crystal	2.0	C
SSO8090	1	**3EXC**	low resolution	2.3	C
YST5158_1_271	1	**3F3K**	Poor crystal	1.8	T
YST4096_1_148	1	**3F4A**	Poor crystal	1.8	T
YST2407_UMP	1	**3F4F**	Poor crystal	2.0	T
YST5764_1_168	3	**3F5R**	No crystal	1.7	C
RHA00566_1_118	1	**3F6O**	No crystal	1.9	Therm
RHA06349_1_131	1	**3F6V**	No crystal	1.5	Pa
ATC0911	1	**3FD3**	No crystal	1.7	V8
APC61717	2	**3FDG**	Poor crystal	1.8	C
ATC1720_1_144	1	**3FHM**	No crystal	2.7	V8
PF1953	1	**3FIO**	No crystal	1.8	C
PF10_0328	8	**3FKM**	No crystal	2.4	C

All structures deposited into the protein databank with corresponding accession code. Resolution numbers are rounded to the nearest tenth of an Angstrom. C = chymotrypsin, T = Trypsin, Therm = Thermolysin, Pa = Papain, V8 = V8 protease.

### Yeast and bacterial proteins

Of the initial 200 yeast and bacterial proteins, two-thirds had previously resisted crystallization, and the remaining third had formed crystals unsuitable for structure determination. These crystals were too small, very thin, formed stacks, or diffracted poorly and could not be improved upon with standard optimization strategies. Occasionally, crystals diffracted well but a very large number of copies in the asymmetric unit (more than 1MD in case of TM1086) made it impractical for structure determination by SAD/MAD techniques. *In situ* proteolysis treatment led to 20 structures from these recalcitrant proteins. Before the *in situ* proteolysis process, 11 had not previously crystallized at all (from ∼135 tested), and the other 9 had formed crystals unsuitable for structure determination (from ∼65 tested). From these data, the technique yields higher success rates within the subset of proteins that had previously formed poor crystals. This trend had been observed in the original study.

#### Human Proteins

Of the 70 human proteins targeted for crystallization by *in situ* proteolysis, 54 had not previously formed crystals, and 16 had formed poor-diffracting crystals. The set that had not formed crystals comprised both those that had not been tested previously as well as a set on which *in situ* proteolysis was performed in parallel with conventional crystallization trials. *In situ* proteolysis treatment led to 12 structures from these recalcitrant proteins. Of these, 5 were from the set of 16 that had previously formed crystals unsuitable for structure determination. This success rate was notably high.

### Parasitic proteins

Of the five proteins from human parasites targeted for crystallization by *in situ* proteolysis, none had previously crystallized. *In situ* proteolysis treatment led to 2 structures.

### Variations on *in situ* proteolysis experimental approaches

The intent of Dong *et al.* was to carry out a systematic, statistically significant, and well-controlled test of the efficacy of *in situ* proteolysis. Each new crystal was analyzed using mass spectrometry to ensure that the success of the method could be attributed directly to the use of proteases. Their paper strongly suggested that the method should be adopted as a primary crystallization strategy due to its high success rate, but was qualified by the fact that the method was being employed in a very controlled setting.

The intent of this study was to examine the efficacy of the method in practice, as carried out on a larger number of proteins by a larger number of experimenters. This strategy had the advantage of exploring the use of the method under less controlled conditions, in which individual investigators adopted slightly different methodologies and strategies. The disadvantage of this strategy is that any conclusions drawn have more caveats, due to the inability to control all aspects of the experiments. We describe here several variations to the original method that have proven successful.

#### *In situ* proteolysis using a wider array of proteases

In the study of 69 target proteins by Dong *et al.*, chymotrypsin was used at a single concentration. In a few cases trypsin was also tested at a single concentration. Chymotrypsin and trypsin are selective proteases: chymotrypsin cleaves on the C-terminal side of bulky hydrophobic side-chains such as phenylalanine, tyrosine, or tryptophan; and trypsin cleaves on the C-terminal side of basic residues. It is likely that many target proteins were not optimal substrates for these enzymes, but in the original study, it was impractical and prohibitively expensive to systematically explore other proteases for the 70 target proteins.

In this study, although chymotrypsin and trypsin accounted for the most successes, other proteases were used, including V8 protease, thermolysin and papain. In total, 26 of the 34 structures derived from the use of chymotrypsin or trypsin, 5 structures derived from the use of the V8 protease, which cleaves on the C-terminal side of acidic residues, two structures derived from the use of thermolysin, and one from papain.

#### Identifying promising proteases in advance of crystallization trials

*In situ* proteolysis was implemented in three different experimental strategies. In the first, exemplified by Dong *et al*, chymotrypsin and/or trypsin were added to the target protein at a fixed concentration. In the second, promising proteases were identified in advance of *in situ* proteolysis, by pre-screening proteases in a range of concentrations and analyzing the results by mass spectrometry or gel electrophoresis. Of the 34 structures, 4 derived from the use of a protease identified in this manner. For example, the structure of N-carbamoylsarcosine amidase from *Thermoplasma acidophilum* was determined after *in situ* trypsinolysis. For this protein, previous attempts at removing the N-terminal 6-His tag with TEV protease were unsuccessful. The seleno-methionine protein was then subjected to limited proteolysis with chymotrypsin, trypsin, V8, and papain prior to analysis by mass spectrometry. Trypsin yielded the largest stable domain and was used for crystallization assays. One condition generated crystals of excellent quality, and the structure was determined at 2.35 Å using SAD from a single data set (Luo *et al.*, in preparation).

The third approach, used largely when the protein was very abundant, is to screen a range of proteases using crystallization, rather than gel electrophoresis, as the metric. This strategy has the advantage that crystallization screens sample a wide range of solution conditions in which the proteases may have more favourable activities. This approach has proven particularly successful for human small GTPases. Commonly, these protein targets were crystallized with four different proteases in parallel – typically chymotrypsin, trypsin, subtilisin, and V8 – at a 1∶100 v/v concentration. The target/protease mixtures were allowed to incubate on ice for thirty minutes, at which point they were subjected to crystallization trials. This technique was applied to 30 different target proteins; 8 structures resulted (PDB codes 3BFN, 3BOR, 3BPJ, 3C5C, 3CBQ, 3CON, 3DZX, and 3EAP), and several more are in process.

### Case studies

#### Sites of cleavage after *in situ* proteolysis

**Figure 1:**
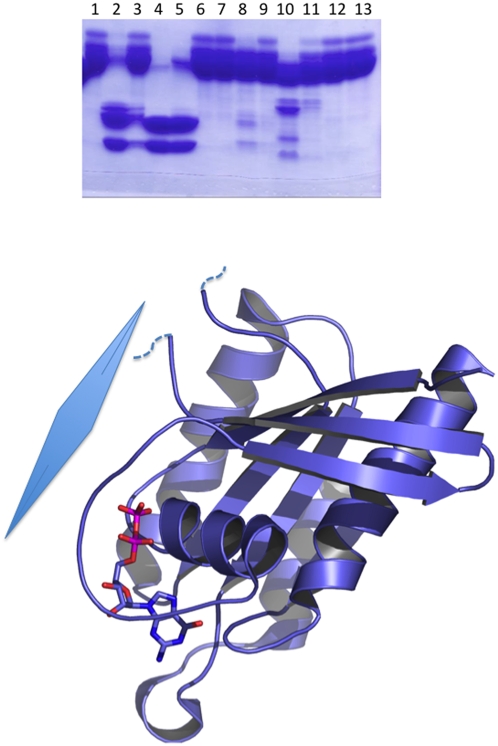
CGD6_3220 proteolytic digestion and subsequent crystal structure. (A) SDS-PAGE gel, lane 1 is untreated protein. Following lanes consist of two concentrations each of chymotrypsin, Trypsin, Elastase, Papain, Subtilisin, and V respectively. Note both chymotrypsin and trypsin yield 2 large stable domains. (B) Ribbon diagram of CGD6_3220. Dotted lines signify missing density, large diamond represents plane of crystal packing.

In most cases described here, as in the study of Dong *et al.*, the cleavage sites appear to reside at the N- and/or C-termini. For instance, the bromodomain of the PF10_0328 protein from *Plasmodium falciparum* would not crystallize using typical crystallization techniques. Eight different constructs were designed and many different protein/crystal screen permutations were tested, with and without tag, and in the presence of six different proteases. Crystals appeared in conditions with chymotrypsin and the structure was solved from selenomethionine-containing protein. From the original construct of 166 residues, including a N-terminal His tag, only 120 residues could be seen in the electron density map. Mass spectrometry analysis of the crystals revealed a large protein peak at 17703 Daltons, which corresponds to chymotrypsin cleavage just C-terminal to Trp463, and no cleavage at the N-terminus (Figure 1).

**Figure 2:**
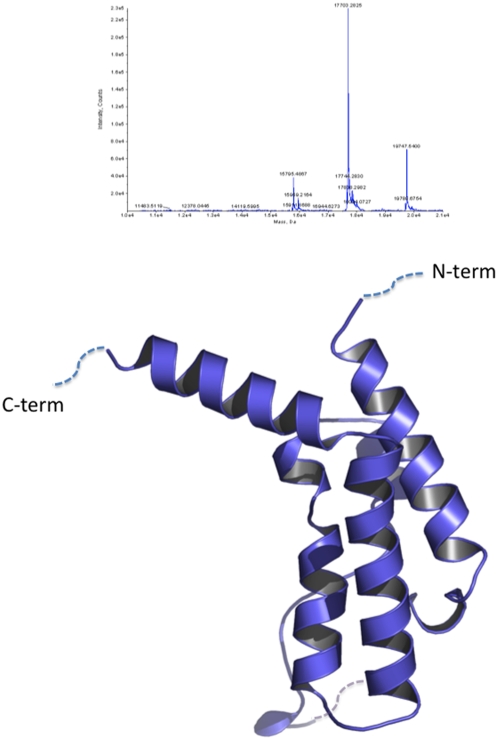
PF10_0328 proteolytic cleavage and crystal structure. (A) Mass spectrometry analysis of chymotrypsin-treated protein crystals. The major peak corresponds to a C-terminal cleavage of 17 residues, largest mass peak corresponds to full length construct, and the third peak corresponds to the C-terminal cleavage plus His-tag cleavage. (B) Ribbon diagram of PF10_0328 bromodomain. Dotted lines signify missing density.

There are a few instances in which the proteins were cleaved at an internal loop. For example, CGD6_3220 is a GTPase from *Cryptosporidium parvum* that failed to crystallize despite repeated attempts with many different constructs. After trypsin treatment, the protein readily crystallized and the structure was solved to 2.2 Å (PDB code 2RHD). All of the protein could be modeled into the electron density map except for one loop corresponding to residues 68–76. Mass spectrometric analysis of the crystals revealed two internal cleavage events at residues 71 and 73, respectively. Interestingly, this loop is located at the interface between two molecules in the crystal lattice (Figure 2). When a highly homologous structure was superimposed on the model and the packing analyzed, the intact loop would have clashed with the symmetry-mate molecule and have inhibited this crystal formation.

#### *In situ* proteolysis removes large amount of protein

As stated earlier, the most common cleavage events occurred near the N and C termini, and usually 20–40 residues were removed. However, in one previously reported case [14] and in one instance reported in this work, a larger polypeptide fragment was removed. For the eukaryotic translation initiation factor 3 (EIF3J; PDB code 3BPJ), 12 different protein constructs were designed in attempts to refine the optimal domain boundaries for successful crystallization. None of the proteins purified from these constructs yielded a diffraction-quality crystal. Chymotrypsin treatment generated a crystal that provided a 1.8 Å dataset from which the structure was solved. The structure revealed that almost half of the protein had been removed; the initial construct contained residues 76–220 but only the C-terminal fragment (141–220) was ordered in the crystal structure. Secondary structure prediction using the JPred server [16] suggested this region was buried, and thus this position would likely not have been selected for construct design.

#### Immediate successes after exhaustive trials

Our default strategy for human proteins is to design 10–15 different constructs for each protein and to attempt to crystallize each one that can be purified, in both the presence and absence of the histidine tag [9]. If this first round fails, more constructs with slight variations at the N- and C-terminal positions are often created, though this strategy is met with significantly diminishing returns. *In situ* proteolysis is now being used to resuscitate some of the failed projects. In one case, 36 constructs were purified for the histone methyltransferase SETDB1 protein, and none crystallized, either with or without the histidine tag. In the first experiment with *in situ* proteolysis, excellent crystals were obtained directly from a matrix screen, and after minor optimization led to crystals diffracting to 1.8 Å (PDB code 3DLM).

The heme-binding transcription factor rev-erbβ and its *Drosophila* orthologue E75 were the subject of extensive efforts over a 2–3 year period. Dozens of constructs, including a number of cysteine mutants and internal deletions, were tested to no avail. Two of the constructs that were expressed to high levels precipitated during concentration for crystallization. A small amount of trypsin was added to facilitate concentration. After successful concentration, the samples were analyzed by denaturing gel electrophoresis, which showed that both proteins had been digested to a single, large, stable domain. Accordingly, *in situ* proteolysis trials with trypsin were pursued with the protein constructs in the presence of the ligand, heme. Initial crystals from a minimal screen were optimized and the protein to protease ratio adjusted, and excellent crystals were obtained from which the structure was solved to 1.9 Å resolution (PDB code 3CQV; Pardee et al, submitted).

Crystallization efforts for the protein TA0507 were initiated in 2001. Several rounds of protein purification and crystallization trials yielded tiny needle clusters that could not be improved in size or quality. The project was abandoned and the protein stored at −80 C for seven years until the *in situ* proteolysis method became more widely used in the laboratory. At that point, the original protein was thawed and chymotrypsin was added. Single crystals were generated from the first crystal screen, and the conditions refined to produce large, single crystals that diffracted to 2.1 Å (PDB code 3DTZ).

#### Reproducibility

Despite initial concerns, crystallization using *in situ* proteolysis appears to be reproducible. In all the experiments reported here, no trouble was observed in reproducing additional crystals for improvement or data collection. On some occasions, the ability to regenerate crystals was remarkable. For example, in the case of SETDB1, the tudor domain of human histone-lysine N-methyltransferase (PDB code 3DLM), excellent native crystals were obtained with chymotrypsin. The experimenter was able to repeatedly obtain crystals with this treatment for extensive testing. Subsequent *in situ* proteolysis trials with the selenomethionine-incorporated protein formed crystals of the same morphology; there was no need to re-test the protease concentration or to re-screen the protein.

#### Alternate protein conformations after *in situ* proteolysis

Crystallization can trap proteins into rare, perhaps non-physiological, conformations; this can also be the case for proteins crystallized after *in situ* proteolysis. The crystallization of rev-erbβ provides such an example. Rev-erbβ crystallized in a number of crystal forms, one of which diffracted to 1.9 Å. In this structure, the heme ligand is coordinated by a histidine residue in the middle of the protein and a cysteine residue in the N-terminal region of the fragment; both residues had been suggested to be ligands for the heme in biochemical and genetic studies [17], [18]. One of the earlier crystal forms of rev-erbβ diffracted to about 3.5 Å. In this crystal form, (Figure S1), the N-terminal portion of the protein had partially unfolded and the protein dimerized around the heme group which was coordinated by the histidine residue in each monomer. The in depth analysis of this crystal form was not pursued, and it is not known if its formation was dependent on proteolysis, or if the conformation represents a physiologically-relevant state.

#### Greater efficacy of method in cases where protein has crystallized previously

The method of *in situ* proteolysis, in its various iterations, has now been applied to over 300 soluble proteins that had previously failed to yield a structure. Of these proteins, ∼200 had never before generated crystals and ∼100 had formed crystals that were unsuitable for structure determination. The success rates of *in situ* proteolysis differed dramatically between these subsets. Of the proteins that had failed to crystallize, 24 structures were obtained, for a success rate of ∼12% (24 structures from 200 proteins). For the proteins that had previously generated crystals, the success rate was almost double at ∼21% (21 structures from 100 proteins).

This large discrepancy between successes of this technique with the two populations could possibly be explained by inherent stability of the purified protein. A protein that had crystallized previously must have been stable enough over the time period required to form crystals. However, the population of proteins that never crystallized might contain a population of unstable proteins, prone to denature or aggregate over the crystallization period. We analyzed possible relationships among the group of proteins that could not crystallize, such as pI or predominant predicted secondary structure, but could not find any common trend among them.

#### Conclusions

In this greater sampling of human and bacterial proteins, *in situ* proteolysis has proven effective when even dozens of constructs failed to produce a protein amenable to crystallization. The method has rescued proteins stored for years and has proven remarkably reproducible. Although we have shown that other proteases can also be used effectively, it is as yet unknown if their use on a large scale is economical, or whether the use of chymotrypsin and trypsin would capture most of the cases that would be successful. The method appears doubly effective when applied to proteins that have already formed crystals as compared to proteins that have never crystallized.

## Supporting Information

Text S1 Original sequences of proteins subjected to *in situ* proteolysis.
(0.05 MB DOC)

Figure S1 Crystal structure figure of a possible non-physiological dimer. Possible non-physiological dimer obtained from a 3.5 Å dataset collected on a crystal of rev-erbβ.
(0.48 MB TIF)
