# Supplementary material for: In Situ Proteolysis to Generate Crystals for Structure Determination: An Update
Source: PLoS One. 2009 Apr 7;4(4):e5094. doi: 10.1371/journal.pone.0005094 (PMC2661377; doi:10.1371/journal.pone.0005094)
Supplement: Text S1 — Original sequences of proteins subjected to in situ proteolysis. (0.05 MB DOC) [file pone.0005094.s001.doc]

Text S1. Original sequences of proteins subjected to in situ proteolysis.

Residues that are missing in 2fofc maps have been underlined.

CGD6_3220: 2RHD

GMNPEYDYLFKLLLIGDSGVGKSCLLLRFADDTYTDSYISTIGVDFKIRTISLENKTVKLQIWDTAGQERFRTITSSYYRGAHGIIIVYDVTDRDSFDNVKQWIQEIDRYAMENVNKLLVGNKCDLVSKRVVTSDEGRELADSHGIKFIETSAKNAYNVEQAFHTMAGEIKKRVQ

KIF22: 3BFN

MHHHHHHSSGRENLYFQGPPARVRVAVRLRPFVDGTAGASDPPCVRGMDSCSLEIANWRNHQETLKYQFDAFYGERSTQQDIYAGSVQPILRHLLEGQNASVLAYGPTGAGKTHTMLGSPEQPGVIPRALMDLLQLTREEGAEGRPWALSVTMSYLEIYQEKVLDLLDPASGDLVIREDCRGNILIPGLSQKPISSFADFERHFLPASRNRTVGATRLNQRSSRSHAVLLVKVDQRERLAPFRQREGKLYLIDLAGSEDNRRTGNKGLRLKESGAINTSLFVLGKVVDALNQGLPRVPYRDSKLTRLLQDSLGGSAHSILIANIAPERRFYLDTVSALNFAARSKEVINRPFTNESLQPHALGPVKLSQKELLGPPEAK

EIF4A2: 3BOR

MHHHHHHSSGRENLYFQGGVIESNWNEIVDNFDDMNLKESLLRGIYAYGFEKPSAIQQRAIIPCIKGYDVIAQAQSGTGKTATFAISILQQLEIEFKETQALVLAPTRELAQQIQKVILALGDYMGATCHACIGGTNVRNEMQKLQAEAPHIVVGTPGRVFDMLNRRYLSPKWIKMFVLDEADEMLSRGFKDQIYEIFQKLNTSIQVVLLSATMPTDVLEVTKKFMRDPIRILVKKE

EIF3J: 3BPJ

mhhhhhhssgrenlyfqgKIAEKIKEKERQQKKRQEEIKKRLEEPEEPKVLTPEEQLADKLRLKKLQEESDLELAKETFGVNNAVYGIDAMNPSSRDDFTEFGKLLKDKITQYEKSLYYASFLEVLVRDVCISLEIDDLKKITNSLTVLCSEKQKQEKQSKAK

BB3683: 3CNV

MGSSHHHHHHSSGRENLYFQGMAEARPDSLTRSRAAKPAGEGAAFSPLYRQIKELLVQSLDRGEWKPGELIPSEIDLAARFQVSQGTVRKAVDELAAEHLLLRRQGKGTFVATHHEARVRYRFLRLAPDEEGEGGRAESRILECRRLRAPAEIARALELRAGETVVTIRRQLSMNHMPTVIDDLWLPGTHFRGLTLELLTASKAPLYGLFESEFGVSMVRADEKLRAVAASPEIAPLLGVEPGRPLLQVDRISYTYGDRPMEVRRGLYLTDHYHYRNSLN

NR1D2-07: 3CQV

MHHHHHHSSGRENLYFQGSSPPSSDFAKEEVIGMVTRAHKDTFMYNQEQQENVPIDGFSQNENKNSYLCNTGGRMHLVCPMSKSPYVDPHKSGHEIWEEFSMSFTPAVKEVVEFAKRIPGFRDLSQHDQVNLLKAGTFEVLMVRFASLFDAKERTVTFLSGKKYSVDDLHSMGAGDLLNSMFEFSEKLNALQLSDEEMSLFTAVVLVSADRSGIENVNSVEALQETLIRALRTLIMKNHPNEASIFTKLLLKLPDLRSLNNMHSEELLAFKVHP

SETDB1: 3DLM

mhhhhhhssgrENLYFQGDLIVSMRILGKKRTKTWHKGTLIAIQTVGPGKKYKVKFDNKGKSLLSGNHIAYDYHPPADKLYVGSRVVAKYKDGNQVWLYAGIVAETPNVKNKLRFLIFFDDGYASYVTQSELYPICRPLKKTWEDIEDISCRDFIEEYVTAYPNRPMVLLKSGQLIKTEWEGTWWKSRVEEVDGSLVRILFLDDKRCEWIYRGSTRLEPMFSMK

DDB1: 3E0C

MSYNYVVTAQKPTAVNGCVTGHFTSAEDLNLLIAKNTRLEIYVVTAEGLRPVKEVGMYGKIAVMELFRPKGESKDLLFILTAKYNACILEYKQSGESIDIITRAHGNVQDRIGRPSETGIIGIIDPECRMIGLRLYDGLFKVIPLDRDNKELKAFNIRLEELHVIDVKFLYGCQAPTICFVYQDPQGRHVKTYEVSLREKEFNKGPWKQENVEAEASMVIAVPEPFGGAIIIGQESITYHNGDKYLAIAPPIIKQSTIVCHNRVDPNGSRYLLGDMEGRLFMLLLEKEEQMDGTVTLKDLRVELLGETSIAECLTYLDNGVVFVGSRLGDSQLVKLNVDSNEQGSYVVAMETFTNLGPIVDMCVVDLERQGQGQLVTCSGAFKEGSLRIIRNGIGIHEHASIDLPGIKGLWPLRSDPNRETDDTLVLSFVGQTRVLMLNGEEVEETELMGFVDDQQTFFCGNVAHQQLIQITSASVRLVSQEPKALVSEWKEPQAKNISVASCNSSQVVVAVGRALYYLQIHPQELRQISHTEMEHEVACLDITPLGDSNGLSPLCAIGLWTDISARILKLPSFELLHKEMLGGEIIPRSILMTTFESSHYLLCALGDGALFYFGLNIETGLLSDRKKVTLGTQPTVLRTFRSLSTTNVFACSDRPTVIYSSNHKLVFSNVNLKEVNYMCPLNSDGYPDSLALANNSTLTIGTIDEIQKLHIRTVPLYESPRKICYQEVSQCFGVLSSRIEVQDTSGGTTALRPSASTQALSSSVSSSKLFSSSTAPHETSFGEEVEVHNLLIIDQHTFEVLHAHQFLQNEYALSLVSCKLGKDPNTYFIVGTAMVYPEEAEPKQGRIVVFQYSDGKLQTVAEKEVKGAVYSMVEFNGKLLASINSTVRLYEWTTEKELRTECNHYNNIMALYLKTKGDFILVGDLMRSVLLLAYKPMEGNFEEIARDFNPNWMSAVEILDDDNFLGAENAFNLFVCQKDSAATTDEERQHLQEVGLFHLGEFVNVFCHGSLVMQNLGETSTPTQGSVLFGTVNGMIGLVTSLSESWYNLLLDMQNRLNKVIKSVGKIEHSFWRSFHTERKTEPATGFIDGDLIESFLDISRPKMQEVVANLQYDDGSGMKREATADDLIKVVEELTRIH

ARHGAP11A: 3EAP

MHHHHHHSSGRENLYFQGMWDQRLVRLALLQHLRAFYGIKVKGVRGQCDRRRHETAATEIGGKIFGVPFNALPHSAVPEYGHIPSFLVDACTSLEDHIHTEGLFRKSGSVIRLKALKNKVDHGEGCLSSAPPCDIAGLLKQFFRELPEPILPADLHEALLKAQQLGTEEKNKATLLLSCLLADHTVHVLRYFFNFLRNVSLRSSENKMDSSNLAVIFAPNLLQTSEGHEKMSSNTEKKLRLQAAVVQTLIDYASDIGRVPDFILEKIPAML

TA0454: 3EEF

MKPALVVVDMVNEFIHGRLATPEAMKTVGPARKVIETFRRSGLPVVYVNDSHYPDDPEIRIWGRHSMKGDDGSEVIDEIRPSAGDYVLEKHAYSGFYGTNLDMILRANGIDTVVLIGLDADICVRHTAADALYRNYRIIVVEDAVAARIDPNWKDYFTRVYGATVKRSDEIEGMLQEDQIET

XCC1504: 3EFG

MHEQLSPRDQELEARLVELETRLSFQEQALTELSEALADARLTGARNAELIRHLLEDLGKVRSTLFADAADEPPPPHY

YST5764: 3F5R

MGSSHHHHHHSSGRENLYFQGMSTDFDRIYLNQSKFSGRFRIADSGLGWKISTSGGSAANQARKPFLLPATELSTVQWSRGCRGYDLKINTKNQGVIQLDGFSQDDYNLIKNDFHRRFNIQVEQREHSLRGWNWGKTDLARNEMVFALNGKPTFEIPYARINNTNLTSKNEVGIEFNIQDEEYQPAGDEGS

RHA00566: 3F6O

MGSSHHHHHHSSGRENLYFQGMAQYPEQLNGIFQALADPTRRAVLGRLSRGPATVSELAKPFDMALPSFMKHIHFLEDSGWIRTHKQGRVRTCAIEKEPFTAVEAWLAEQQELWESRTDRLEQFVTEHTVTAHAKATPQ

RHA06349: 3F6V

MGSSHHHHHHSSGRENLYFQGHMNSPTSRPLRRDPLHNALVTTNVLVVLDQLEVAAEPTRRRLVQLLTSGEQTVNNLAAHFPASRSAISQHLRVLTEAGLVTPRKDGRFRYYRLDPQGLAQLRALFDSFWIDELDRLVADATEEAASKGDS

ATC0911: 3FD3

mvcsgapvaliptssgaimldypsmravalvartgsfekaaqvlcvtpsavsqrikqleerlgvvlivrgnpcvatekgewlcrhmdhvgmleselfrqlpalteagdaqervtlniatnadslgtwfldavskftggsdylvniavddqdhtvewlrggrvlaavtahdkpvqgcrvtplgvlryhataspdfmarhfadgvtpaalarapgltfnqkdrlqaswirtalgedvsypthwlpstdgfvkaslagmgwglnpvqlvaehlaagrlvelmpgtpldiplywqvnrlaaerlagltanmvgtarvvlmpvg

PF1953: 3FIO

MAQKILVEQVVKRKAIVVQPKDTVDRVAKILSRNKAGSAVVMEGDEILGVVTERDILDKVVAKGKNPKEVKVEEIMTKNPVKIEYDYDIEDVIELMTEKGVRRVLVTKFGKPIGFVTAADILAALASHNHEEEEEEREEESEVYGICEVCGQYGALYKVYHEGRELWVCETCKDLIEGR

APC85800.6: 3DOA

MAYDGLFTKKMVESLQFLTTGRVHKINQPDNDTILMVVRQNRQNHQLLLSIHPNFSRLQLTTKKYDNPFNPPMFARVFRKHLEGGIIESIKQIGNDRRIEIDIKSKDEIGDTIYRTVILEIMGKHSNLILVDENRKIIEGFKHLTPNTNHYRTVMPGFNYEAPPTQHKINPYDITGAEVLKYIDFNAGNIAKQLLNQFEGFSPLITNEIVSRRQFMTSSTLPEAFDEVMAETKLPPTPIFHKNHETGKEDFYFIKLNQFNDDTVTYDSLNDLLDRFYDARGERERVKQ

PF10_0328: 3fkm

MHHHHHHSSGRENLYFQGNKQWYLLANQLILSLSKYEGGHIFEKLVDAKKQNCPDYYDVIKNPMSFSCIKTKLKKGQYAYPSEFVKDVQLIFDNCSLYNTSNSVVAITGKNIETYFNNQLIVMGYNNFILKEKKINDMLKLVEEENIKWSKEKEDQIEEIDELENN

ATC1720: 3FHM

MGSSHHHHHHSSGRENLYFQGMATFVKDLLDRKGRDVVTVGPDVSIGEAAGTLHAHKIGAVVVTDADGVVLGIFTERDLVKAVAGQGAASLQQSVSVAMTKNVVRCQHNSTTDQLMEIMTGGRFRHVPVEENGRLAGIISIGDVVKARIGEIEAEAEHIKAYIAG

RASL12: 3C5C

MHHHHHHSSGRENLYFQGPLEVNLAILGRRGAGKSALTVKFLTKRFISEYDPNLEDTYSSEETVDHQPVHLRVMDTADLDTPRNCERYLNWAHAFLVVYSVDSRQSFDSSSSYLELLALHAKETQRSIPALLLGNKLDMAQYRQVTKAEGVALAGRFGCLFFEVSACLDFEHVQHVFHEAVREARRE

EPHA2: 3C8X

APEHHHHHHDYDIPTTENLYFQGAMDAAQGKEVVLLDFAAAGGELGWLTHPYGKGWDLMQNIMNDMPIYMYSVCNVMSGDQDNWLRTNWVYRGEAERIFIELKFTVRDCNSFPGGASSCKETFNLYYAESDLDYGTNFQKRLFTKIDTIAPDEITVSSDFEARHVKLNVEERSVGPLTRKGFYLAFQDIGACVALLSVRVYYKKCP

ATC2088: 3DNH

GHMLDVAPPVITPRGTKIEPSAGAPFEAVRVARDVLHTSRTAALATLDPVSGYPYTTATNIGIEPDGTPFFFAAGLTLHARNMETDARISVTLAPFGKGDALTLPRLTLVGRADRIGPDEVPLAIARYIARYPKAKLYLSLPDTRLYRLRTEGVQINGGPARNASNITPADLRTDLSGAEELMAAAESEATRLNAIKGEASRLAVLAGAKTGRWKITSIDPDGIDLASASDLARLWFAERVETLKQFEKALAQLLKGS

TA0507: 3DTZ

MGSSHHHHHHSSGLVPRGSHMTEIYTSVLSYRLLEGKAYSDADTRSLDRMMRSIDEFFSANPGYINFHIYRSYRTDSDVIFWYSSRNPDLMILAKERVQASMRPIAVSSFSSISIYDESPYNAMNKKLEDSLRLPPLRYFVAYPMSKTPDWYLLDFDTRKEIMHEHIKMALNHPDEKGIRSYTTYSFGIGDQEFVVLYEIPDIAAWSRVTEKLREARARKWIIKETPILLGRLVDAGDIAGFLL

YST5158: 3F3K

mpsltprciivrhgqtewsksgqytgltdlpltpygegqmlrtgesvfrnnqflnpdnityiftsprlrarqtvdlvlkplsdeqrakirvvvdddlreweygdyegmltreiielrksrgldkerpwniwrdgcengettqqiglrlsraiariqnlhrkhqsegrasdimvfahghalryfaaiwfglgvqkkcetieeiqnvksydddtvpyvklesyrhlvdnpcflldaggigvlsyahhnidepalelagpfvsppegs

YST4096: 3F4A

MGSSHHHHHHSSGRENLYFQGMDSYSITNVKYLDPTELHRWMQEGHTTTLREPFQVVDVRGSDYMGGHIKDGWHYAYSRLKQDPEYLRELKHRLLEKQADGRGALNVIFHCMLSQQRGPSAAMLLLRSLDTAELSRCRLWVLRGGFSRWQSVYGDDESVTAGYLPDLWR

YST2407_ump: 3F4F

MGSSHHHHHHSSGLVPRGSHMTATSDKVLKIQLRSASATVPTKGSATAAGYDIYASQDITIPAMGQGMVSTDISFTVPVGTYGRIAPRSGLAVKNGIQTGAGVVDRDYTGEVKVVLFNHSQRDFAIKKGDRVAQLILEKIVDDAQIVVVDSLEESARGAGGFGSTGN

APC61717: 3FDG

mtetipvfdghndfllrllrnpanretiwlkgdgtghldlprmkeggfaggffaiyvpspqahdaahfeammdappfelplppmiraeqaqpvalamaghllwmeraargrfkvcrtaaevrschadgivsgimhmegaeaigadldalhlfhslglrslgpvwsrptvfghgvpfrfpgspdtgeglteagrrlvaecnrlkimldlshlnekgfddvarlsdaplvathsnahavtpstrnltdrqlamiresrgmvglnfatsflredgrrsaemgwepvlrhldhlidrlgedhvgmgsdfdgatipqgiadvtglpalqaamrahgydeplmrklchenwygllerswga

APC7436: 3EEt

MGSSHHHHHHSSGRENLYFQGHMTFGEQPAYLRVAGDLRKKIVDGSLPPHTRLPSQARIREEYGVSDTVALEARKVLMAEGLVEGRSGSGTYVRERPVPRRVARSGYRPDSGATPFRQEQADGAVRGTWESHSEQAEASGAIAERLDIRPGERVMCTKYVFRDAGEVMMLSTSWEPLAVTGRTPVMLPEEGPVGGMGVVERMAAIDVIVDNVTEEVGARPGLAEELLTLGGVPGHVVLVIQRTYFASGRPVETADVVVPADRYRVAYHLPVK

TM1086: 3DCL

GHMRTNKDRLVRISVVGEIAPAKMRSPYSVTTEGTVRVIPVLGGITYNVKVGDSAYGWAGDHVEPGVSVMARRKEEEIPLMTLSCIGNEVIVMSGDAKGSRGFVTGKHGGVNHVLVHFEEEVLGKLMVGDKILIKAWGQGLKLLDHPDVKVMNIDPDLFEKLGIQEKNGKIHVPVVAKIPAHMMGSGIGASSSASTDYDIMASNPEDLGVADLKLGDIVAIQDHDNSYGVGKYRKGAVSIGVVVHSACVSAGHGPGVVVIMTGDESKILPEEVERANISDYLVR

REM2: 3CBQ

MHHHHHHSSGRENLYFQGQKDGIFKVMLVGESGVGKSTLAGTFGGLQGDSAHEPENPEDTYERRIMVDKEEVTLVVYDIWEQGDAGGWLRDHCLQTGDAFLIVFSVTDRRSFSKVPETLLRLRAGRPHHDLPVILVGNKSDLARSREVSLEEGRHLAGTLSCKHIETSAALHHNTRELFEGAVRQIRLRRGRNHA

NRAS: 3CON

MHHHHHHSSGRENLYFQGMTEYKLVVVGAGGVGKSALTIQLIQNHFVDEYDPTIEDSYRKQVVIDGETCLLDILDTAGQEEYSAMRDQYMRTGEGFLCVFAINNSKSFADINLYREQIKRVKDSDDVPMVLVGNKCDLPTRTVDTKQAHELAKSYGIPFIETSAKTRQGVEDAFYTLVREIRQYRMKKLN

AF0924: 3DT5

GHSNRQVQLMARQQRLKAIEDRLEKFYIPLIKAFSSYVYTAQTEDEIETIITCRRYLAGNNLLRVLPMHFKFKADKIAGSANWTFYAKEDFEQWKEALDVLWEEFLEVLKEYYTLSGTEISLPEKPDWLIGYKGS

TBC1D22B: 3DZX

MHHHHHHSSGRENLYFQGMTVREKTRLEKFRQLLSSQNTDLDELRKCSWPGVPREVRPITWRLLSGYLPANTERRKLTLQRKREEYFGFIEQYYDSRNEEHHQDTYRQIHIDIPRTNPLIPLFQQPLVQEIFERILFIWAIRHPASGYVQGINDLVTPFFVVFLSEYVEEDVENFDVTNLSQDMLRSIEADSFWCMSKLLDGIQDNYTFAQPGIQKKVKALEELVSRIDEQVHNHFRRYEVEYLQFAFRWMNNLLMRELPLRCTIRLWDTYQSEPEGFSHFHLYVCAAFLIKWRKEILDEEDFQGLLMLLQNLPTIHWGNEEIGLLLAEAYRLKYMFADAPNHYRR

SS08090: 3EXC

MHHHHHHSSGRENLYFQGMKLLVVYDVSDDSKRNKLANNLKKLGLERIQRSAFEGDMDSQRMKDLVRVVKLIVDTNTDIVHIIPLGIRDWERRIVIGREGLEEWLV

Figure S1. Possible non-physiological dimer obtained from a 3.5 Å dataset collected on a crystal of rev-erbβ.
