# Supplementary figures and images for: In Situ Proteolysis to Generate Crystals for Structure Determination: An Update
Source: PLoS One. 2009 Apr 7;4(4):e5094. doi: 10.1371/journal.pone.0005094 (PMC2661377; doi:10.1371/journal.pone.0005094)

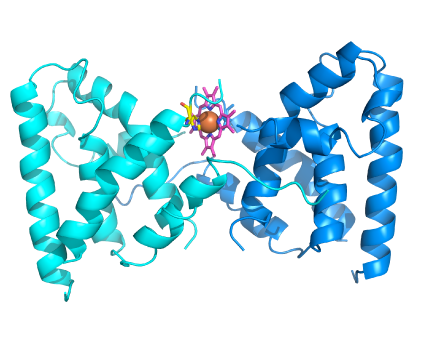

Supplement: Figure S1 — Crystal structure figure of a possible non-physiological dimer. Possible non-physiological dimer obtained from a 3.5 Å dataset collected on a crystal of rev-erbβ. (0.48 MB TIF) [file pone.0005094.s002.tif]
